# Clinical manifestations and cerebrospinal fluid status in ocular syphilis in HIV-Negative patients

**DOI:** 10.1186/s12879-016-1586-z

**Published:** 2016-06-06

**Authors:** Ting Dai, Xinjun Wu, Shaona Zhou, Qianqiu Wang, Daning Li

**Affiliations:** Department of Dermatology, Shanghai Xuhui Central Hospital, Shanghai, People’s Republic of China; Department of Neurology, Shanghai Xuhui Central Hospital, Shanghai, People’s Republic of China; Department of Clinical Management, National Center for STD Control, Nanjing, Jiangsu People’s Republic of China

**Keywords:** Cerebrospinal fluid, HIV-negative, Ocular syphilis

## Abstract

**Background:**

Syphilis with ocular involvement has reemerged as a critical health problem. The aim of the present study was to explore the clinical manifestations and cerebrospinal fluid (CSF) status in ocular syphilis in human immunodeficiency virus (HIV)-negative patients.

**Methods:**

The clinical records of patients with ocular syphilis presenting to the Shanghai Xuhui Central Hospital in the period from January 2011 to December 2012 were retrospectively reviewed.

**Results:**

The median age of 25 HIV-negative patients with ocular syphilis was 53 years, 18 patients (72.0 %) were males and 7 (28.0 %) were females. None of them self-identified themselves as men who had sex with men (MSM). The ocular lesions included: uveitis (13 cases), optic neuropathy (6 cases), retinal vasculitis (5 cases), retinal detachment (3 cases), and neuroretinitis (4 cases). Serum toluidine red unheated serum test (TRUST) titer ranged from 1 to 512, with a median of 64. Overall, 18 (72.0 %) of the 25 patients had abnormal CSF results, 15 (60.0 %) CSF samples had elevated white blood cell counts, 13 (52.0 %) had elevated protein levels, and 9 (36.0 %) had reactive CSF Venereal Disease Research Laboratory (VDRL) test, respectively. Mann–Whitney U tests showed higher serum TRUST titer (>32) correlated with the abnormal CSF results.

**Conclusions:**

The demographic characteristics of patients with ocular syphilis in this study were different from previous reports. The study showed a high CSF abnormal rate in HIV-negative patients. The recommendation for CSF examination from all patients with ocular syphilis, including HIV-negative cases, is strongly supported by the present data.

## Background

Syphilis is a chronic sexually transmitted disease caused by a spirochete *Treponema pallidum* subsp. *pallidum* that can affect most organ systems, including the eyes. Syphilis has been considered as “the great masquerader” and often presents with manifestations similar to various other ocular diseases. It can involve all parts of the eye and may be the only manifestation of syphilis. In the pre-antibiotic era, syphilis was recognized as one of the most common causes of intraocular inflammation. The introduction of penicillin during the 1950s led to a decrease in the incidence of syphilis; hence, vigilance was required in the diagnosis of ocular syphilis [1, 2].

Even with no clinical evidence of central nervous system (CNS) disease, the US Centers for Disease Control and Prevention (CDC) recommended a standard regimen of intravenous penicillin for neurosyphilis at a dose of 18–24 million units daily for 10–14 days [3]. Outside of case reports or small case series, little is known about the concomitant CNS involvement in ocular syphilis. A significantly higher proportion of human immunodeficiency virus (HIV)-positive patients with ocular syphilis compared with HIV-negative cases have been reported in published studies [4–8]. In a meta-analysis in HIV-positive patients with ocular syphilis, 74 % had elevated cerebrospinal fluid (CSF) white blood cell (WBC) count, 75 % had elevated CSF protein while 57 % had positive CSF Venereal Disease Research Laboratory (VDRL) test [9]. Few data exist about the CSF status in ocular syphilis in HIV-negative individuals. HIV increases the risk for both neurosyphilis and CSF abnormalities [3]; therefore important questions remain about the relationship between ocular syphilis and neurosyphilis. Uncertainties exist in the optimal management of ocular manifestations of syphilis due to a lack of evidence.

The prevalence of syphilis has increased sharply in the last decade in several countries around the world, including China. Since the invention of penicillin, no country has experienced such a dramatic upsurge of reported syphilis cases, as China is presently having [10]. Nationwide surveillance data indicated that the incidence of syphilis in the general population increased from 0.17 cases per 100,000 in 1993 to 20.0 cases per 100,000 in 2010 [11]. Syphilis has now become the most commonly reported communicable disease in Shanghai, the largest city in China, with 76.42 cases per 100,000 reported in 2011 [11, 12]. Syphilis with ocular involvement has reemerged as a critical health problem. A failure to recognize the ocular manifestations of syphilis or delayed diagnosis and treatment can lead to irreversible visual loss. For a better understanding of HIV-negative patients with ocular syphilis, especially CSF status, a case series is presented here.

## Methods

### Study participants

Located in Shanghai, the Eye & ENT (Eye, Ear, and Throat) Hospital of Fudan University is the biggest Eye and ENT hospital in east China. Patients who visited the hospital with eye, ear or throat-related symptoms suspected of syphilis were suggested to have a blood screening for syphilis, and those screened positive were transferred to Shanghai Xuhui Central Hospital (Shanghai Clinical Center, Chinese Academy of Science) for further treatment. All the patients with ocular syphilis presenting to Shanghai Xuhui Central Hospital were advised to have CSF examination. The clinical records of patients with ocular syphilis in the period from January 2011 to December 2012 were retrospectively reviewed. Individuals were eligible for enrollment in this study if they 1) presented initially with ocular symptoms 2) had serological evidence of syphilis and 3) had completed CSF examination. The collected data included chief complaint, demographic data, cocurrent medical problems, ophthalmologic findings, syphilis serology, HIV serology and CSF examination results. Written informed consents were obtained from all participants for lumbar puncture according to the routine medical procedure.

### Laboratory methods

*Treponema pallidum particle agglutination* (TPPA) tests were conducted using the Serodia-TPPA reagent from Fujirebio Diagnostics Inc (Malven, Pennsylvania, USA). The results were observed according to the manufacturer’s instructions. Serum toluidine red unheated serum test (TRUST) and CSF VDRL tests were performed according to the standard methods. The TRUST reagents were manufactured by Shanghai Rongsheng Biotech, and the VDRL antigen with buffered saline were manufactured by Becton-Dickinson and Company (Diagnostic Systems, MD, USA). All patients underwent HIV screening through a dual enzyme-linked immunosorbent assay.

### Statistical methods

Subjects with reactive CSF TPPA were classified as having abnormal CSF if they also met one of the following criteria: reactive CSF VDRL, elevated CSF WBC (>5 cells/mm3) count, or elevated protein level (>45 mg/dL). Qualitative variables were described using frequencies and percentages, while quantitative variables were described using medians and interquartile ranges (IQR). Mann–Whitney *U*-test was used to compare variables between abnormal and normal CSF subjects. Data were recorded using Microsoft Excel, and analyses were performed using Statistical Package for the Social Sciences (SPSS15.01, SPSS Inc.,, IL, USA).

### Ethics statement

The Ethical Committee of the Shanghai Xuhui Central Hospital (Shanghai Clinical Center, Chinese Academy of Science) waived the need for institutional review board approval because of the retrospective nature of this study. The participants were not required to provide written informed consent for their clinical records to be used in this study. Patient information was anonymized and de-identified prior to analysis. The described research adhered to the principles of the Declaration of Helsinki.

## Results

During the 2-year period, 25 patients with syphilis were identified who initially presented with ocular symptoms and completed CSF examination. All patients were treated unsuccessfully with nonspecific therapy for ocular symptoms before the correct diagnosis was made. None of them had a previous history of syphilis. The interval between ocular symptom onset and the first visit at the hospital ranged from 2 weeks to 20 years (median 4 months, IQR 13 month). The median age at presentation was 53 years (range 30–77 years). All 25 patients were of Han ethnicity. Eighteen patients (72.0 %) were males, and seven were females (28.0 %). Twenty-four patients (17 males and 7 females) identified themselves as heterosexual; another male refused to provide sexual partner information. One male and three female patients got syphilis infections from their spouses. Of the 25 patients, 3 (12 %) had mucocutaneous manifestation of secondary syphilis, 5 (20 %) had a history of papulomacular rash on the trunk and palms or soles of the feet, 2 (8 %) reported previous genital ulcers, and 15 (60 %) had no systemic signs. Ocular symptoms were bilateral in 16 (64 %) cases. None of the 25 patients tested positive for HIV infection. The ocular lesions included: uveitis (52 %,13 cases), optic neuropathy (24 %, 6 cases), retinal vasculitis (20 %, 5 cases), retinal detachment (12 %, 3 cases), and neuroretinitis (16 %, 4 cases). Six (24 %) patients had multiple ocular lesions. Patients with comorbidities included hypertension (20 %, five cases), diabetes mellitus (24 %, six cases), tuberculosis infection (4 %, one case), hepatitis C (4 %, one case), renal atrophy (4 %, one case), cancer (4 %, one case), and hypercholesterolemia (8 %, two cases). Clinical and CSF features of the 25 patients are presented in Table 1.

**Table 1:** Demographic, clinical, and laboratory data of patient with ocular syphilis

Case	Age/Sex	Serum TRUST	CSF- VDRL	CSF- WBC cells/mm^33^	CSF- Protein mg/dL	Ocular findings
1	M	128	N	11	1.44	Neuroretinitis
2	M	16	4	2	0.58	Retinal vasculitis
3	M	64	2	1	0.68	Uveitis, optic neuropathy
4	F	8	N	0	0.23	Uveitis, Neuroretinitis
5	F	16	4	1	0.50	Uveitis
6	F	2	N	0	0.31	Uveitis, retinal vasculitis
7	M	512	4	30	0.4	Uveitis
8	M	128	N	8	0.49	Neuroretinitis
9	M	4	N	3	0.32	Optic neuropathy
10	M	64	N	24	0.46	Retinal detachment
11	M	32	1	15	0.51	Uveitis
12	M	32	N	20	0.54	Uveitis
13	F	2	N	4	0.27	Uveitis
14	M	16	1	23	0.17	Retinal vasculitis
15	F	64	8	64	0.62	Uveitis, optic neuropathy
16	F	128	N	18	0.41	Uveitis, Neuroretinitis
17	M	1	N	4	0.37	Retinal detachment
18	M	4	N	4	0.30	Uveitis
19	M	64	N	12	0.54	Uveitis
20	M	4	N	0	0	Retinal vasculitis
21	M	64	N	41	0.286	Retinal detachment
22	F	64	N	29	0.53	Optic neuropathy
23	M	128	1	10	0.48	Optic neuropathy
24	M	128	N	16	0.3	Uveitis
25	M	64	8	154	0.66	Optic neuropathy

All patients had positive serum TPPA and TRUST results. The range of serum TRUST titer ranged from 1 to 512, with a median of 64. Overall, 18 (72.0 %) of the 25 patients had abnormal CSF results. Of which, 15(60.0 %) CSF samples had elevated WBC counts, 13(52.0 %) had elevated protein level, 9(36.0 %) had reactive CSF VDRL test, respectively. The CSF cell count ranged from 0 to 154, with a median of 11cells/mm^3^ (normal ≤5 cells/mm^3^). The CSF protein level ranged from 17 to 144, with a median of 46 mg/dl (normal ≤45 mg/dl). The CSF VDRL titer ranged from 0 to 8.

Mann–Whitney *U*-test revealed that a higher serum TRUST titer (>32) correlated with the abnormal CSF results (*P* < 0.05). The other investigated factors including age, gender, and interval between ocular disease onset and the diagnosis were not associated with ocular syphilis (Table 2).

**Table 2:** Variables associated with abnormal CSF

	Normal CSF	Abnormal CSF	
Categorical variables	Freq (%)	Freq (%)	*P*
Gender			0.355
Male (*n* = 18)	4 (22.2)	14 (77.8)	
Female (*n* = 7)	3 (42.9)	4 (57.1)	
Age			0.378
≤ 50 (*n* = 10)	4(40.0)	6 (60.0)	
> 50 (*n* = 15)	3 (20.0)	12 (80.0)	
Basic Serum TRUST			<0.001
≤ 32(*n* = 12)	6 (50.0)	6 (50.0)	
> 32(*n* = 13)	1 (7.7)	12 (92.3)	
Duration			0.550
≤ 3 month (*n* = 12)	3 (25.0)	9 (75.0)	
> 3 month (*n* = 13)	4 (30.8)	9 (69.2)	

## Discussion

During the 1960s to 1980s, no new case of ocular syphilis was reported in China. However, dozens of cases with ocular syphilis were reported from a single urban setting in the past two years. This is in accordance with recent syphilis epidemic in China [11, 12].

Most studies on ocular syphilis originated in the Western world, and only a few studies have reported on the features of this disease in Asian patients. In previous reports from the United States, United Kingdom, France and other Western Europe countries, most of the increase in the incidence of syphilis occurred among men, especially young men who had sex with men (MSM) and was associated with the HIV epidemic [13–17]. Although most patients here were males, none of them indentified themselves as MSM, except for one male who refused to provide sexual orientation; 12 of the 18 acknowledged a history of unprotected intercourse with female sex workers. Most of the patients in this study were in their fifties to sixties, our recent national survey among male clients with sexually transmitted diseases in China has also shown that elder patients were more likely to have syphilis infection [18]. Older patients of low socio-economic status may be more likely to visit low-fee female sex workers and less likely to use condoms. Furthermore, unlike most reports from Western countries, none of the patients with ocular syphilis were HIV-positive in this study. The result is in agreement with other reports from China;despite a high syphilis rate in some high-risk populations, the HIV infection rate is relatively low [19, 20]. Demographic characteristics of ocular syphilis in the present study were substantially different compared with other reports. The patients in this cohort seemed to be more atypical. If patients are presumed to be in low-risk groups, delays in diagnosis and therapy may be likely.

It is recommended that all patients with ocular complications of syphilis involvement undergo a lumbar puncture. However, most of the previous reports from ophthalmologists were focused on ocular findings. No large studies have been conducted to determine the incidence of concomitant CNS involvement in patients with ocular syphilis. Case series from the pre-HIV era and pooled data in HIV-positive patients suggested that most patients with ocular syphilis had an elevated WBC count or total protein in the CSF, which is consistent with the results of this study [9, 21]. Conflicting data exist about the CSF VDRL results among patients with ocular syphilis. Although up to 54 % HIV-positive patients showed reactive VDRL in pooled analysis, Spoor’s study from the pre-HIV era found that none of the 50 individuals with ocular syphilis had a reactive CSF VDRL [9, 21]. In the present study, all the patients were HIV-negative, and nearly one third had a reactive CSF-VDRL test. It is not surprising that HIV-positive patients had a higher abnormal CSF rate than in HIV-negative patients, since co-infection with HIV could alter the severity of syphilis and increases the likelihood of syphilitic CNS involvement. The discrepancy in CSF characteristics of HIV-negative patients between Spoor’s study and the present results could be explained as follows: Frist, the patient in this study had a higher serum non-*treponemal* test (100 % had reactive serum TRUST, median titer 64) compared with the Spoor’s report (only 24 % had reactive serum VDRL, titer up to 16) [21]. Second, our previous study showed that the predominant *Treponema pallidum* genotype in Shanghai was *14d/f* (88.8 %), which was suspected to be a high-risk neurosyphilis genotype [22].

The present study found that patients with ocular syphilis having a higher serum TRUST titer (>32) significantly associated with an abnormal CSF. Other investigated factors including age, gender, and interval between ocular disease onset and the diagnosis were not associated with ocular syphilis. These findings are consistent with the previous reports that found a relationship between higher serum non-treponemal test (≥32) and neurosyphilis [22]. This might better explain why the present study had a higher CSF-VDRL compared with the Spoor’s study.

This study had several limitations. First, the clinical data were collected from a single center and the study was retrospective, therefore, both selection and information biases should be acknowledged. Second, the sample size was relatively small, limiting the ability to control confounding variables and perform multiple statistical analyses. Third, syphilis has no pathognomonic ocular presentation. Polymerase chain reaction of aqueous and vitreous humor, can help in making an accurate diagnosis, and hence may be performed in future studies.

## Conclusions

The present study showed a high CSF abnormal rate in HIV-negative patients with ocular syphilis. The recommendation for CSF examination from all patients with ocular syphilis including HIV-negative cases is strongly supported by the present data. More research is needed to better understand ocular syphilis among HIV-negative patients.

## Abbreviations

CDC, US Centers for Disease Control and Prevention; CNS, central nervous system; CSF, cerebrospinal fluid; ENT, Eye, Ear and Throat; HIV, human immunodeficiency virus; MSM, men who have sex with men; TPPA, *Treponema pallidum* particle agglutination; TRUST, Toluidine red unheated serum test; VDRL, Venereal Disease Research Laboratory; WBC, white blood cell
